# Pharmacological recapitulation of the lean phenotype induced by the lifespan-extending sulfur amino acid-restricted diet

**DOI:** 10.18632/aging.206237

**Published:** 2025-04-07

**Authors:** Naidu B. Ommi, Dwight A.L. Mattocks, Karel Kalecký, Teodoro Bottiglieri, Sailendra N. Nichenametla

**Affiliations:** 1 Animal Science Laboratory, Orentreich Foundation for the Advancement of Science Inc., Cold Spring-on-Hudson, NY 10516, USA; 2 Center of Metabolomics, Institute of Metabolic Disease, Baylor Scott and White Research Institute, Dallas, TX 75204, USA

**Keywords:** buthionine sulfoximine, thiols, serine, anti-obesity drugs, aging

## Abstract

Sulfur amino acid restriction (SAAR), lowering the dietary concentration of sulfur amino acids methionine and cysteine, induces strong anti-obesity effects in rodents. Due to difficulties in formulating the SAAR diet for human consumption, its translation is challenging. Since our previous studies suggest a mechanistic role for low glutathione (GSH) in SAAR-induced anti-obesity effects, we investigated if the pharmacological lowering of GSH recapitulates the lean phenotype in mice on a sulfur amino acid-replete diet. Male obese C57BL6/NTac mice were fed high-fat diets with 0.86% methionine (CD), 0.12% methionine (SAAR), SAAR diet supplemented with a GSH biosynthetic precursor, N-acetylcysteine in water (NAC), and CD supplemented with a GSH biosynthetic inhibitor, DL-buthionine-(S, R)-sulfoximine in water (BSO). The SAAR diet lowered hepatic GSH but increased Nrf2, Phgdh, and serine. These molecular changes culminated in lower hepatic lipid droplet frequency, epididymal fat depot weights, and body fat mass; NAC reversed all these changes. BSO mice exhibited all SAAR-induced changes, with two notable differences, i.e., a smaller effect size than that of the SAAR diet and a higher predilection for molecular changes in kidneys than in the liver. Metabolomics data indicate that BSO and the SAAR diet induce similar changes in the kidney. Unaltered plasma aspartate and alanine transaminases and cystatin-C indicate that long-term continuous administration of BSO is safe. Data demonstrate that BSO recapitulates the SAAR-induced anti-obesity effects and that GSH plays a mechanistic role. BSO dose-response studies in animals and pilot studies in humans to combat obesity are highly warranted.

## INTRODUCTION

Age-related dysregulation of lipid metabolism contributes to diabetes, obesity, and metabolic syndrome. It also increases the risk for cardiovascular diseases, cancers, arthritis, and Alzheimer’s disease [1]. Prevention and treatment of lipid metabolic dysfunction improves overall healthspan and lifespan. Existing treatment modalities are disease-specific, moderately effective, do not cause complete remission, and exert various adverse effects [2]. More importantly, drugs targeting common underlying mechanisms are highly desirable due to the comorbidity of metabolic disorders. Well-established laboratory interventions extending overall metabolic healthspan and lifespan are ideal paradigms for identifying such common mechanisms.

Laboratory studies suggest restricting the dietary concentration of sulfur amino acids methionine and cysteine (SAAR) protects against multiple diseases, including obesity, diabetes, cardiovascular diseases, cancers, and Alzheimer’s [3–7]. The anti-obesity effect of the SAAR diet is so potent that it decreases the body weights of obese mice by 50% within three weeks and confers complete resistance to body fat accretion, even on a 60% kcal fat diet [8, 9]. The SAAR diet confers these benefits despite *ad libitum* dietary intake. Encouraged by laboratory findings, we and others conducted human studies with the best possible formulations of the SAAR diet as a potential therapeutic strategy for metabolic diseases [4, 10–12]. However, findings from these studies indicate that the changes in humans were not as robust as in rodents. These studies also suggest that formulating the SAAR diet for human consumption and long-term adherence to such a diet will be challenging [13]. A more feasible approach would be inducing the SAAR diet-associated signaling mechanisms through drugs. While multiple studies identified SAAR-induced changes in lipogenic and lipolytic genes and proteins that alter lipid metabolism, the exact biochemical underpinnings that initiate these changes remain unknown [14]. Identifying such biochemical pathways provides new targets to improve human healthspan.

We reported that low tissue glutathione (GSH) levels might be mechanistically associated with the SAAR-induced lean phenotype [15]. GSH is a tripeptide of glutamic acid, cysteine, and glycine. Cysteine, the most limiting amino acid in the diet for GSH biosynthesis, is eliminated from the SAAR diet [16, 17]. Since GSH is essential, rodents on the SAAR diet synthesize it by converting some of the available methionine to cysteine through transsulfuration. However, due to low dietary methionine levels, total GSH (tGSH = reduced GSH + oxidized GSH) concentrations in the livers and kidneys of animals on the SAAR diet range from 25% to 50% of those on the control diet [18, 19]. A primary consequence of low tGSH is the induction of Nrf2, a transcription factor associated with antioxidant defenses and energy metabolism [20, 21]. We reported that the SAAR-induced Nrf2 increases serine biosynthesis by diverting substrates from glyceroneogenesis, which is required for triglyceride recycling. This substrate paucity eventually results in lower body adiposity. By supplementing the SAAR diet with progressively increasing concentrations of cysteine, we demonstrated that all these changes were reversed, starting with an increase in tGSH concentrations in a dose-dependent manner. These studies were conducted on a diet with standard fat content in non-obese rats; hence, the relevance of these findings in the context of obesity and on a high-fat diet remains unknown.

In the current study, we investigated if pharmacological lowering of GSH alters body composition in the context of a high-fat diet (60% kcal from fat) by inducing the Nrf2/Phgdh/serine pathway. Four groups of diet-induced obese mice were treated with combinations of two diets and two drugs. The first two groups received high-fat diets with high (CD, control diet) and low methionine (SAAR diet) concentrations. These two groups were not treated with any drugs. The third group received the SAAR diet but was supplemented with N-acetylcysteine (NAC), which increases tissue GSH concentrations. The fourth group was fed the CD diet and treated with DL-buthionine-(S, R)-sulfoximine (BSO), which inhibits GSH synthesis.

## MATERIALS AND METHODS

### Animal husbandry

All animal procedures were approved by the Institutional Animal Care and Use Committee of the Orentreich Foundation for the Advancement of Science, Inc. (Protocol No. 04-2022). Eleven-week-old male diet-induced obese C57BL/6NTac mice (n=28) were purchased from Taconic Biosciences (Model No. DIO-B6-M, Germantown, NY, USA). Mice were acclimatized to the new housing and environmental conditions of 20 ± 2° C, 50 ± 10% relative humidity, and a 12:12 h light/dark cycle for seven weeks. During acclimatization, mice were fed a high-fat control diet with 60% kcal from fat and 0.86% w/w methionine without cysteine (Catalog No. A14032002, Research Diets, New Brunswick, NJ, USA). At 18 weeks old, mice were quasi-randomized into four intervention groups, each with similar average body weights (n=7/group). Two groups were fed diets with control (CD – 0.86% w/w methionine without cysteine) and restricted sulfur amino acid concentrations (SAAR - 0.12% w/w methionine without cysteine). These two groups did not receive any pharmaceutical compounds in water. To investigate if increasing GSH concentration reverses the effects of the SAAR diet, a third group was fed the SAAR diet but supplemented with NAC, a precursor of GSH biosynthesis. To investigate if the pharmacological lowering of GSH induces similar changes as the SAAR diet, we fed a fourth group (BSO) with the CD diet and supplemented water with BSO, an inhibitor of GSH biosynthesis. The CD and SAAR diets were isonitrogenous and isocaloric. To balance the decrease in total nitrogen due to low methionine in the SAAR diet, glutamic acid was increased. Detailed composition of both diets is provided in Supplementary Table 1. Diets were offered *ad libitum* and with continuous access. CD and SAAR groups received *ad libitum* water. NAC and BSO groups received *ad libitum* water with 30 mM NAC (Catalog No. A9165, Sigma Aldrich, St. Louis, MO, USA) and 30 mM BSO (Catalog No. B690250, Toronto Research Chemicals Inc., Toronto, ON, Canada), respectively. Animals were housed singly with weekly changes in food, water, bedding, and cages throughout the 13-week intervention. Body weight, body composition and food and water intakes were monitored weekly.

On the day of sacrifice, blood glucose concentrations were determined from the tail-clips using a FreeStyle Lite glucometer (Abbott Laboratories, Abbott Park, IL, USA). Just after that, food was withdrawn for three hours starting at 8:00 AM, and the blood was collected from the retro-orbital sinus of anesthetized mice. Plasma was obtained by centrifuging the blood at 9000 xg for 3 min at 4° C. After sacrificing the mice by cervical dislocation, the liver, kidney, and epididymal adipose depot were harvested, weighed, immediately flash-frozen, and stored at -80° C. A piece of liver was incubated overnight with RNA*later* (Catalog No. AM7021, ThermoFisher Scientific, Waltham, MA, USA) at 4° C. The next day, RNA*later* was discarded, and the tissues were stored at -80° C. An aliquot of liver and epididymal adipose tissue was fixed in 10% neutral buffered-formalin overnight; later, the formalin was discarded, and the tissues were stored in 70% ethanol at 4° C.

### Glutathione, cysteine, serine, and methionine quantification

The liver and kidney were homogenized in 10 to 15 volumes of 5% ice-cold metaphosphoric acid and incubated on ice for 10 min. The homogenates were centrifuged at 10,000 xg for 5 min at 4° C. Supernatants were separated into new Eppendorf tubes and stored at -80° C until used.

Glutathione in the supernatants was quantified using the modified Tietze method described elsewhere [19, 22–24]. Total cysteine (tCys = reduced cysteine + oxidized cysteine) was quantified using the acid ninhydrin reagent following the procedure described by Gaitonde [25]. Cystine and mixed disulfides of cysteine in the samples were reduced to cysteine before quantifying for tCys. Supernatants (125 μL) were incubated in glass tubes with an equal volume of 10 mM DTT and 100 μL of 1N NaOH at room temperature for 30 min. The reaction mixture was acidified with 250 μL of glacial acetic acid and 250 μL of freshly prepared ninhydrin reagent (625 mg ninhydrin, 15 mL acetic acid, and 10 mL concentrated HCl). The tubes were heated in a boiling water bath for 10 min and rapidly cooled under tap water, after which 500 μL of 95% ethanol was added to the reaction. The concentration of the resulting cysteine-ninhydrin conjugate in the sample was determined by measuring the absorbance at 570 nm and comparing it with the absorbance of known standards. tGSH and tCys concentrations were expressed as mg/g wet tissue. Methionine and serine concentrations were obtained from targeted metabolomics analysis (described in section “Metabolomics analysis”).

### Protein isolation and immunoblotting

Liver and kidney tissues were homogenized in ice-cold RIPA buffer containing protease/phosphatase inhibitors (Catalog No. 5872, Cell Signaling, Danvers, MA, USA), sonicated for 30 sec, and incubated on ice for 30 min. After incubation, homogenates were centrifuged at 10,000 xg for 10 min at 4° C, and the supernatants were stored at -80° C. Proteins were electrophoresed on 4-20% Criterion™ TGX™ Precast Midi Protein gels (Bio-Rad Laboratories, Hercules, CA, USA) at 200 V. Proteins separated on the gels were transferred onto PVDF membranes and blocked with either 5% nonfat dry milk powder (Catalog No. 1706404, Bio-Rad Laboratories, Hercules, CA, USA) or BSA (Catalog No. 9998, Cell Signaling Technology, Danvers, MA, USA) in TBS containing 0.1% Tween 20 (TBST 0.1%) for one hour at room temperature. After blocking, PVDF membranes were incubated with primary and secondary antibodies for target proteins and loading controls. Antibody sources and incubation conditions are provided in Supplementary Table 2. Clarity™ Western ECL Blotting Substrates (Catalog No. 1705061, Bio-Rad Laboratories, Hercules, CA, USA) or SuperSignal™ West Femto Maximum Sensitivity Substrate (Catalog No. 34096, ThermoFisher Scientific, Waltham, MA, USA) were used to develop the bands on the membrane. The protein bands were visualized using the ChemiDoc XRS+ system (Bio-Rad Laboratories, Hercules, CA), and the band intensities were quantified using Image Lab software (Bio-Rad Laboratories, Hercules, CA, USA). β-Actin and vinculin were used as loading controls. If membranes were to be used to re-probe for a second target protein, they were stripped using Restore Western Blot Stripping Buffer (Catalog No. 21059, Thermo Fisher Scientific, Waltham, MA, USA) at 37° C for 15 min.

### Histological analysis

Lipid droplet frequency in the liver and adipocyte area in the epididymal adipose depot were quantified. A 4 μm section was made from paraffin-embedded tissues fixed in 10% neutral-buffered formalin and stained with hematoxylin and eosin. Images were acquired using a Leica AT2 and GT450 scanner (Leica Biosystems, Nussloch, Germany). The number of lipid droplets was determined in five non-overlapping fields per section at 10X magnification. The cell counter plugin in ImageJ software (NIH, Bethesda, MD, USA) was used to count the number of droplets. Data were expressed as the average number of droplets per field.

Hematoxylin and eosin-stained images of epididymal adipose tissue sections were acquired at 20X magnification from five different fields. The adipocyte area was quantified using ImageJ software. The scale was calibrated using the image scale bar length and setting the unit of length as a micrometer (μm). The area of each adipocyte was calculated by drawing a border around each cell using the freehand selection tool and expressed as μm^2^.

### Body composition

Body composition, including fat and lean mass, was determined using a magnetic resonance whole-body composition analyzer (EchoMRI™-900V, Echo Medical Systems, Houston, TX, USA). Mice were weighed and moderately restrained in a plastic probe (EchoMRI-A100™) inserted into the scanner for 3 min. Data were reported as g of fat mass and lean mass.

### Gene expression

Total RNA was isolated from the RNA*later*-treated liver by homogenizing them in TRIzol™ reagent (Catalog No. 15596018, Invitrogen, Waltham, MA, USA). The homogenate was separated into aqueous and organic phases by adding chloroform, and the total RNA in the aqueous phase was precipitated by adding 100% isopropanol. RNA was pelleted by centrifuging the isopropanol mixture at 12,000 xg for 10 min at 4° C. Pellets were washed with 75% ethanol, air-dried under the hood for 15 minutes, and dissolved in RNase-free water. RNA concentration and purity were estimated using a NanoDrop 2000c spectrophotometer. Residual DNA was digested by treating 2.5 μg of RNA with DNase-I (Catalog No. 18068015, Invitrogen, Waltham, MA, USA) for 15 min at room temperature; the enzyme was later inactivated by adding 25 mM EDTA for 10 min at 65° C. RNA was reverse transcribed using a high-capacity cDNA reverse transcription kit (Catalog No. 4374966, Applied Biosystems, Waltham, MA, USA). The cDNA obtained was diluted 10-fold for use in gene expression assays. Real-time PCR was performed in a StepOnePlus Real-Time PCR system (Applied Biosystems, Waltham, MA, USA) using TaqMan chemistry. All TaqMan assays spanned exon-exon junctions, and *Ppib* was used as the internal control in all assays. Details of the TaqMan assays are provided in Supplementary Table 3. The comparative ΔΔC_T_ method was used to determine the changes in gene expression, and the fold change in expression was derived using the formula 2^(-ΔΔCT)^.

### Metabolomics analysis

Targeted metabolomics was performed using a Sciex 7500 QTrap mass spectrometer (AB Sciex LLC, Framingham, MA, USA) coupled to a Shimadzu Nexera chromatography system (Shimadzu Corporation, Kyoto, Japan). The Biocrates MxP Quant 500 XL targeted metabolomics kit (Biocrates Life Sciences AG, Innsbruck, Austria) was used according to the manufacturer’s instructions. Frozen liver and kidney aliquots were homogenized and deproteinized by sonication with three volumes of isopropyl alcohol. Following centrifugation, 10 μL of this extract from each sample was applied to each 96-well plate from the Quant 500 XL kit. The plate contained isotope-labeled internal standards and included wells for blanks, seven-point calibrators, and 3 levels of quality control samples. Mass spectrometry intensities were acquired in Sciex Analyst c1.6.24 (AB Sciex LLC, Framingham, MA, USA). Raw chromatographic signal files were processed in the in-house software Integrator to obtain intensity areas of measured metabolites and their area ratios with the respective internal standards. Data were preprocessed as described in detail elsewhere [26].

### Plasma markers of liver and kidney damage

Plasma markers of liver damage (aspartate aminotransferase [AST] and alanine transaminase [ALT]) and kidney damage (cystatin-C) were quantified using enzyme-linked immunosorbent assay (ELISA) kits. All assays were performed according to the manufacturer’s instructions. All samples were analyzed in duplicates, and the concentrations were derived by interpolating the sample absorbance against a standard curve. Details of ELISA kits are provided in Supplementary Table 4.

### Statistics

All data were analyzed using GraphPad Prism. A one-way analysis of variance, followed by Tukey’s multiple comparison test, was used to find pair-wise differences. Differences were considered statistically significant if P-values were <0.05. Pair-wise differences were reported by asterisks (* for P ≤ 0.05, ** for P ≤ 0.01, *** for P ≤ 0.001, and **** for P ≤ 0.0001). Although pair-wise comparisons were done all-way, only four comparisons relevant to our hypothesis are shown in the figures. Occasionally, a two-tailed Student’s t-test was used if Tukey’s test did not yield significant P-values despite large mean differences; P-values from the t-test were indicated by “#.” The sample size was seven per group in most analyses, but it was slightly lower for some because of outliers or lack of data due to insufficient biospecimens. All outliers were determined using Grubb’s test.

To detect metabolites affected by the interventions, we performed differential analysis as a series of Welch’s t-tests, one for each metabolite, between the control group and each treatment group. Subsequently, for each treatment group, the two-tailed P-values across the tests were controlled for false discovery rate (FDR) using the q-value procedure with R package “qvalue” [27, 28]. The FDR control was done separately for each analytical method (UHPLC/FIA). Differences with FDR ≤ 0.05 were considered statistically significant. Principal component analysis (PCA) was performed with the R package “ropls” with an automatically fitted number of principal components [28]. The resulting principal components were used to detect changes between individual groups with the ZWL test implemented in the R package “highDmean” [28, 29]. P-values were adjusted for multiple testing with Holm’s method [30]. Sample heatmaps show the top 50 metabolites with the most significant group differences according to ANOVA P-values.

## RESULTS

### BSO and the SAAR diet induce similar morphometric changes

**Figure 1:**
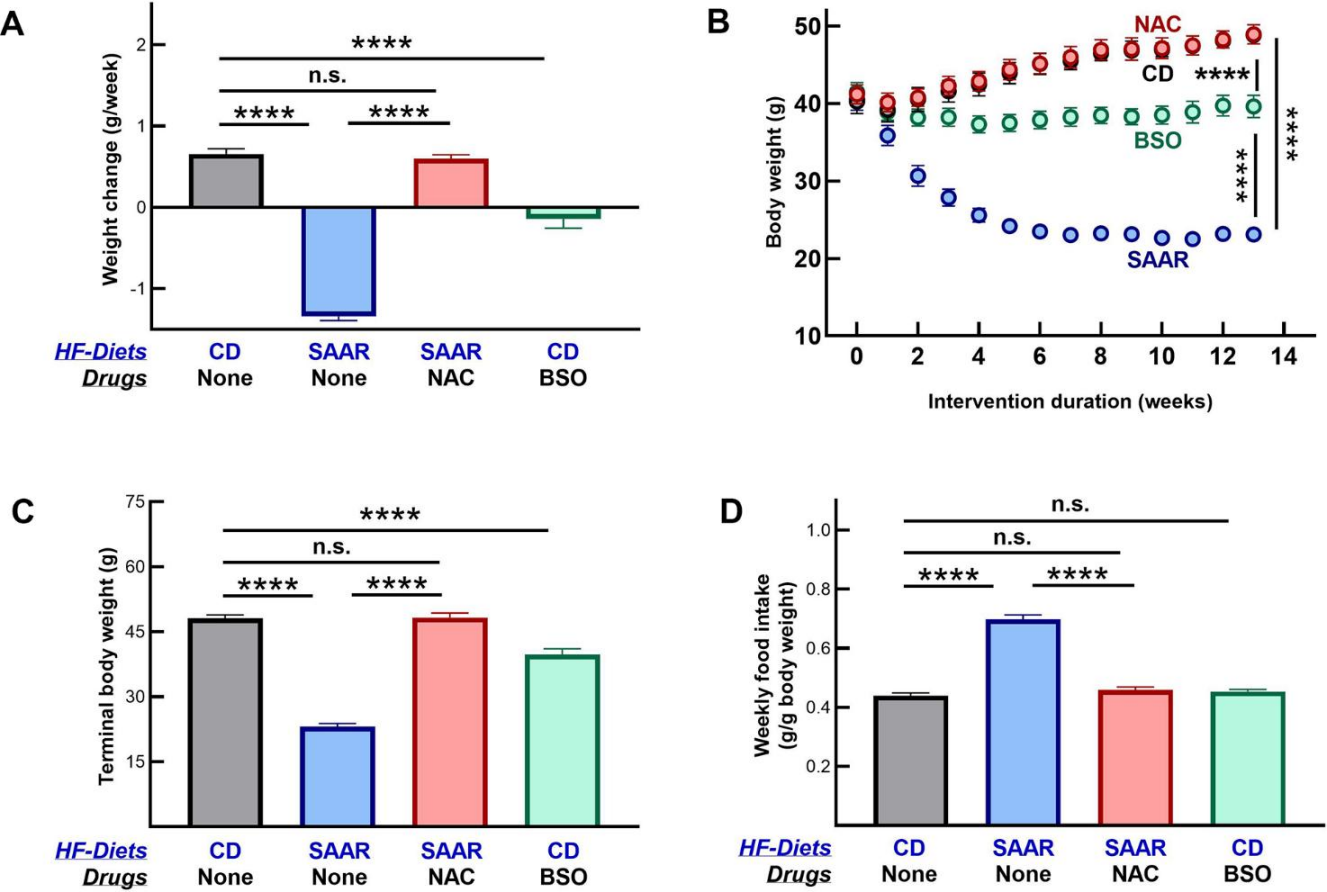
**The SAAR diet and BSO induce similar morphometric changes.** Four groups of eighteen-week-old male C57BL/6NTac mice (n=7/group) were fed high-fat (60% Kcal from fat) diets with 0.86% w/w methionine (CD), 0.12% w/w methionine (SAAR), SAAR diet with 30 mM N-acetylcysteine in water (NAC), and CD diet with 30 mM DL-buthionine (S, R) sulfoximine (BSO). Compared to the CD, the SAAR diet caused weight loss (**A**–**C**) and increased food intake (**D**). Despite feeding on the SAAR diet, mice in the NAC group did not exhibit any changes (**A**–**D**). Except for the lack of increased food intake, changes in BSO mice were similar to those in the SAAR mice, despite feeding on a diet replete with sulfur amino acids (**A**–**D**). Note: One-way ANOVA followed by Tukey’s multiple comparison tests was used to find group-wise differences (*P ≤ 0.05, **P ≤ 0.01, ***P ≤ 0.001, ****P ≤ 0.0001. Bars and error bars represent means and standard error of means). CD growth curves are invisible as NAC growth curves overlay them.

Mice on CD grew at 0.65±0.06 g/week, while those on the SAAR diet lost weight at 1.34±0.05 g/week (Figure 1A). The weight gain in CD was gradual throughout the intervention, while the weight loss in the SAAR mice was rapid and occurred only in the first five weeks (Figure 1B). By the end of the study, SAAR mice (23.19±0.63 g) weighed 47% of CD mice (48.09±0.81 g, Figure 1C). As reported in our earlier studies, mice on the SAAR diet lost weight despite the higher food intake on a body weight basis (Figure 1D) [31, 32]. The weekly food consumption in the SAAR mice (0.69±0.01 g/g body weight) was 160% of that in the CD mice (0.43±0.008 g/g body weight, Figure 1D).

NAC supplementation reversed all SAAR effects, i.e., CD and NAC mice had similar body weights, growth rates, and food consumption (Figure 1A–1D). Morphometric changes in BSO mice were in the same direction as in the SAAR mice but with a smaller effect size. Compared to the baseline, BSO mice neither lost nor gained body weight during the study (Figure 1A, 1B). However, due to the weight gain in CD mice, the terminal body weights in the BSO mice (39.73±1.33) were 81% of that in CD mice (48.09±0.81g, Figure 1C). Unlike the SAAR diet, BSO did not increase food intake (Figure 1D).

### BSO and the SAAR diet exert tissue-specific effects on tGSH concentrations and Nrf2/Phgdh/serine pathway

**Figure 2:**
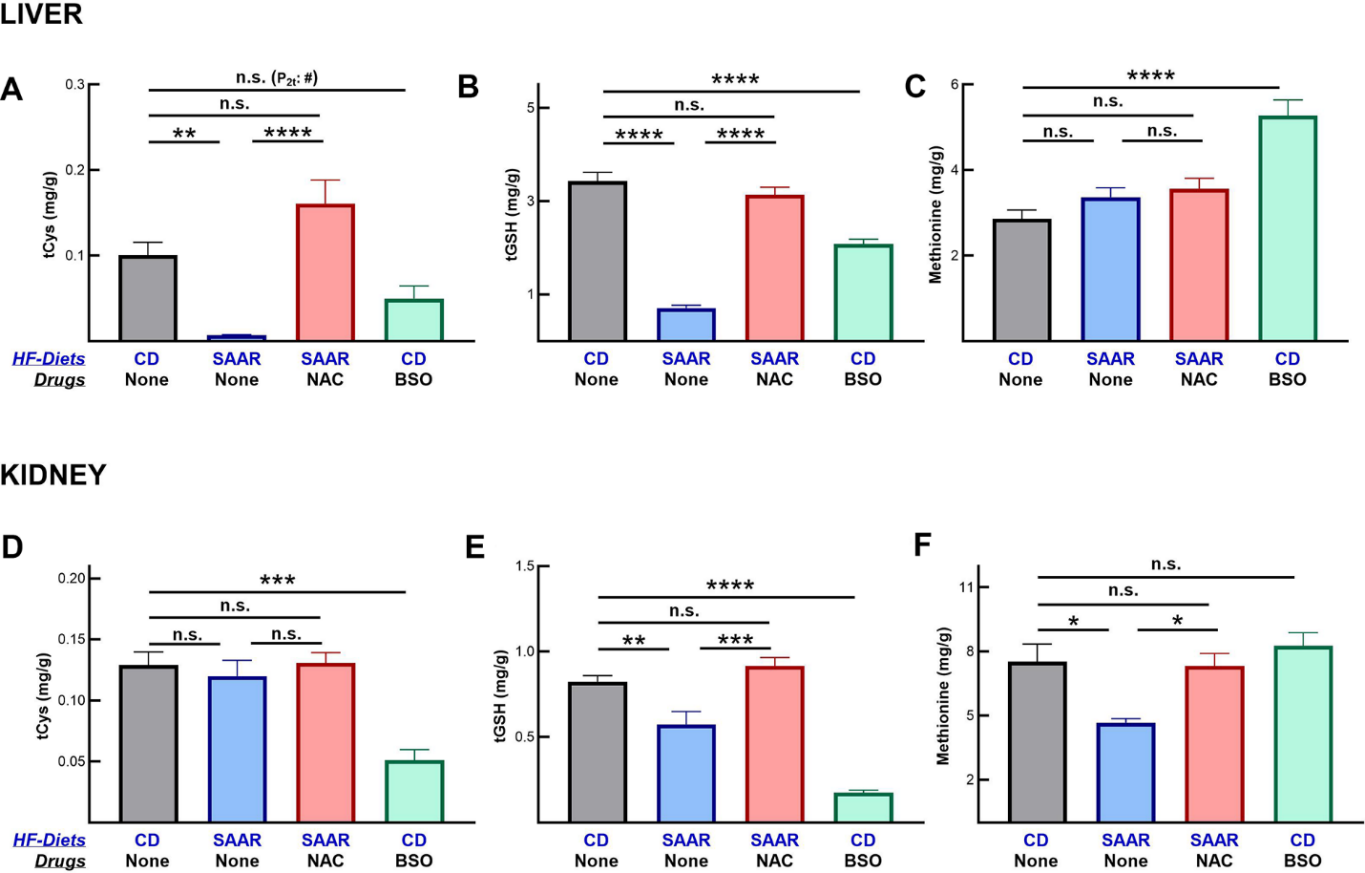
**BSO decreases tissue concentrations of cysteine and glutathione, but not of methionine.** Similar to the SAAR diet, BSO decreased total cysteine (tCys) (**A**) and total glutathione (tGSH) (**B**) in the liver, but to a lower extent. Unlike the SAAR diet, which did not alter hepatic methionine, BSO increased it (**C**). BSO exerted stronger effects than the SAAR diet on tCys (**D**) and tGSH (**E**) in the kidney. The SAAR diet, but not BSO, decreased methionine in the kidney (**F**). NAC reversed the impact of the SAAR diet on both sulfur compounds. Note: # indicates P-values from a 2-tailed Student’s t-test; the rest of the statistical methods are similar to those in Figure 1.

Similar to our earlier reports, the SAAR diet decreased hepatic concentrations of tCys and tGSH (Figure 2A, 2B) [15, 19]. Like the SAAR diet, BSO also decreased the hepatic concentrations of these compounds. However, in BSO mice the magnitude of the decrease in the hepatic tCys (SAAR - 7% of CD and BSO - 49% of CD) and tGSH (SAAR - 20% of CD and BSO - 60% of CD) was lower compared to the decreases in the SAAR mice. Despite an 80% decrease in dietary methionine, hepatic methionine concentrations were unaltered in SAAR mice (Figure 2C). Previous studies corroborate our data on hepatic methionine [33, 34]. Unlike the SAAR diet, BSO increased hepatic methionine concentrations (180% of CD, Figure 2C).

Contrary to its milder effect than the SAAR diet in the liver, BSO exerted a stronger effect on the renal concentrations of tCys (SAAR - 93% of CD, BSO - 39% of CD, Figure 2D) and tGSH (SAAR - 69% of CD, BSO - 21% of CD, Figure 2E). NAC supplementation reversed the effects of the SAAR diet both in the livers and kidneys. The SAAR diet, but not BSO, decreased methionine concentration in the kidney (62% of CD, Figure 2F).

**Figure 3:**
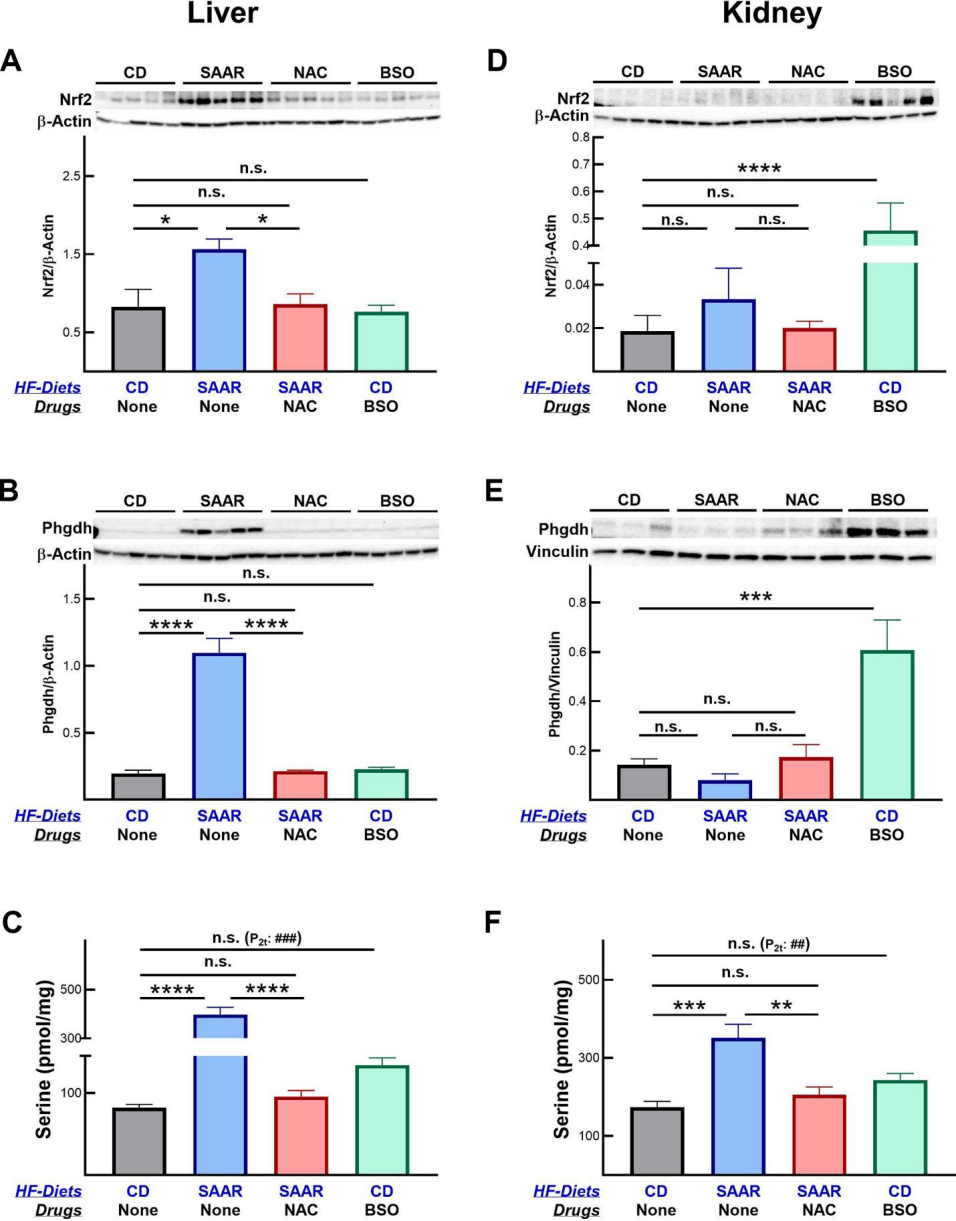
**The SAAR diet and BSO exert tissue-specific effects on Nrf2 and Phgdh.** The SAAR diet increased Nrf2 (**A**) and Phgdh (**B**) protein expressions in the liver, which ultimately resulted in higher serine concentrations (**C**). Unlike the SAAR diet, BSO did not increase Nrf2 and Phgdh in the liver but increased both in the kidneys (**D**, **E**). Regardless of the changes in Nrf2 and Phgdh, BSO increased serine concentrations in livers and kidneys (**C**–**F**). NAC reversed SAAR-induced changes in Nrf2, Phgdh, and serine (**A**–**F**). Note: Sample size = 5-6. Statistical methods are similar to those in Figure 2.

Consequent to the changes in hepatic and renal concentrations of tGSH, the SAAR diet and BSO exerted tissue-specific effects on Nrf2 and Phgdh protein expressions (Figure 3). The effects of these interventions on the protein expression were restricted to the tissues on which they exerted maximal effects. Despite a significantly lower hepatic tGSH, BSO did not increase Nrf2 and Phgdh in the liver (Figure 3A, 3B). However, BSO increased both Nrf2 (2451% of CD) and Phgdh (427% of CD) in the kidneys (Figure 3D, 3E), where the decrease in tGSH (21% of CD) was higher than that in the liver (60% of CD) (Figure 2B, 2E). Similarly, the effect of the SAAR diet on these proteins was restricted to the liver (Figure 3A, 3B), as the decrease in the hepatic tGSH (20% of CD) was greater than that in the kidney (69% of CD) (Figure 2B, 2E). Based on these data, we speculate that the induction of Nrf2 and Phgdh by low tissue tGSH might be contingent upon decreasing it to levels below a certain threshold. Despite the strong effects of the SAAR diet on the hepatic tGSH, Nrf2, and Phgdh expression, all these effects were reversed with the supplementation of NAC. Regardless of the tissue-specific increases in Nrf2 and Phgdh, both interventions increased hepatic serine and the increase was greater in the SAAR mice (485% of CD) than in the BSO mice (163% of CD, Figure 3C). Like its effect on hepatic serine, BSO also increased renal serine (140% of CD, Figure 3C, 3F). Previous studies reported the export of serine from the kidney into the liver and its anti-adiposity effects [35]. Whether such inter-organ transport occurs in the BSO-treated mice in our study remains to be investigated. As in the case of Nrf2 and Phgdh protein expression, NAC supplementation also reversed the increase in tissue serine concentration by the SAAR diet.

### Similar to the SAAR diet, BSO exerts anti-obesity effects

**Figure 4:**
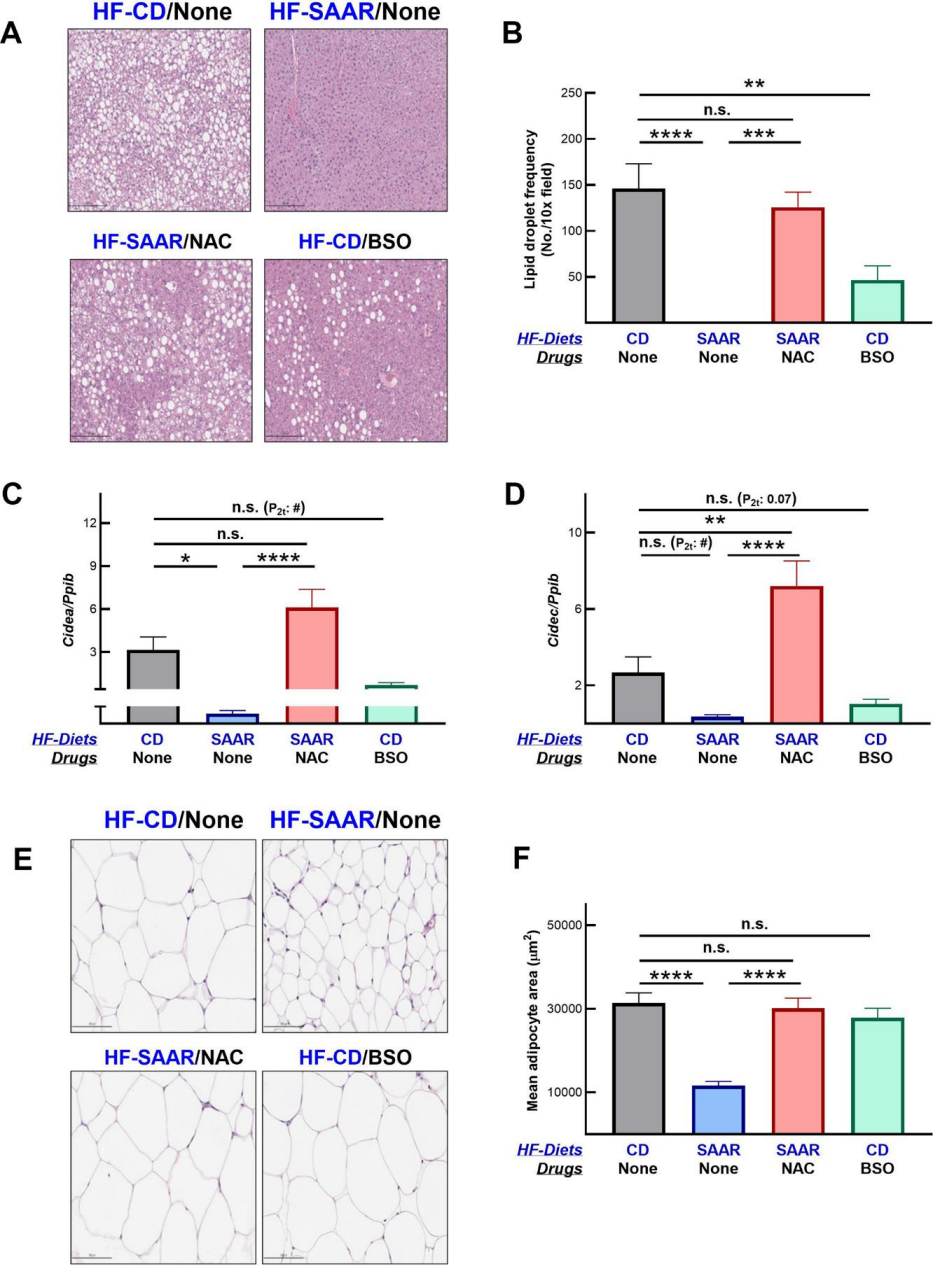
**BSO decreases hepatic lipid droplet frequency.** Hematoxylin and eosin-stained liver sections show a lower frequency of lipid droplets in mice on BSO than in CD but higher than those on the SAAR diet (**A**). A bar graph of lipid droplet quantification is shown in (**B**). Gene expression data show that both the SAAR diet and BSO decrease the expression of *Cidea* (**C**) and *Cidec* (**D**), proteins involved in lipid droplet synthesis. Unlike the SAAR diet, BSO did not decrease the adipocyte area in epididymal adipose tissue (**E**, **F**). NAC reversed all the SAAR-induced changes (**A**–**F**). Note: Statistical methods are similar to those in Figure 2. The sample size was 6-7/group.

As expected, the SAAR diet strongly decreased hepatic lipid droplet frequency (Figure 4A, 4B). The effect was so strong that we did not observe any lipid droplets in the hepatic sections of mice on the SAAR diet. Despite such a strong effect by the SAAR diet, NAC supplementation completely reversed the lipid droplet frequency, almost to the levels observed in the CD mice. These data, combined with the lack of changes in hepatic methionine concentrations, demonstrate that the anti-adiposity effects of the SAAR diet are specifically due to low levels of tGSH and/or its constituent amino acid tCys but not due to low methionine. Although not as strong as the SAAR diet, BSO decreased the lipid droplet frequency in mice even on the CD diet (31% of CD, Figure 4A, 4B). Decreases in the hepatic lipid droplet frequency were paralleled in the expression of genes involved in lipid droplet synthesis. The SAAR diet strongly inhibited the expression of *Cidea* (0.2% of CD, Figure 4C) and *Cidec* (13% of CD, Figure 4D), while NAC completely reversed such an effect. Akin to the SAAR diet, BSO repressed the expression of *Cidea* (22% of CD, Figure 4C) and *Cidec* (38% of CD, Figure 4D); the statistical significance of the BSO effect on these genes was marginal (Cidea - P_2t_ -0.01; Cidec - P_2t_ - 0.07).

**Figure 5:**
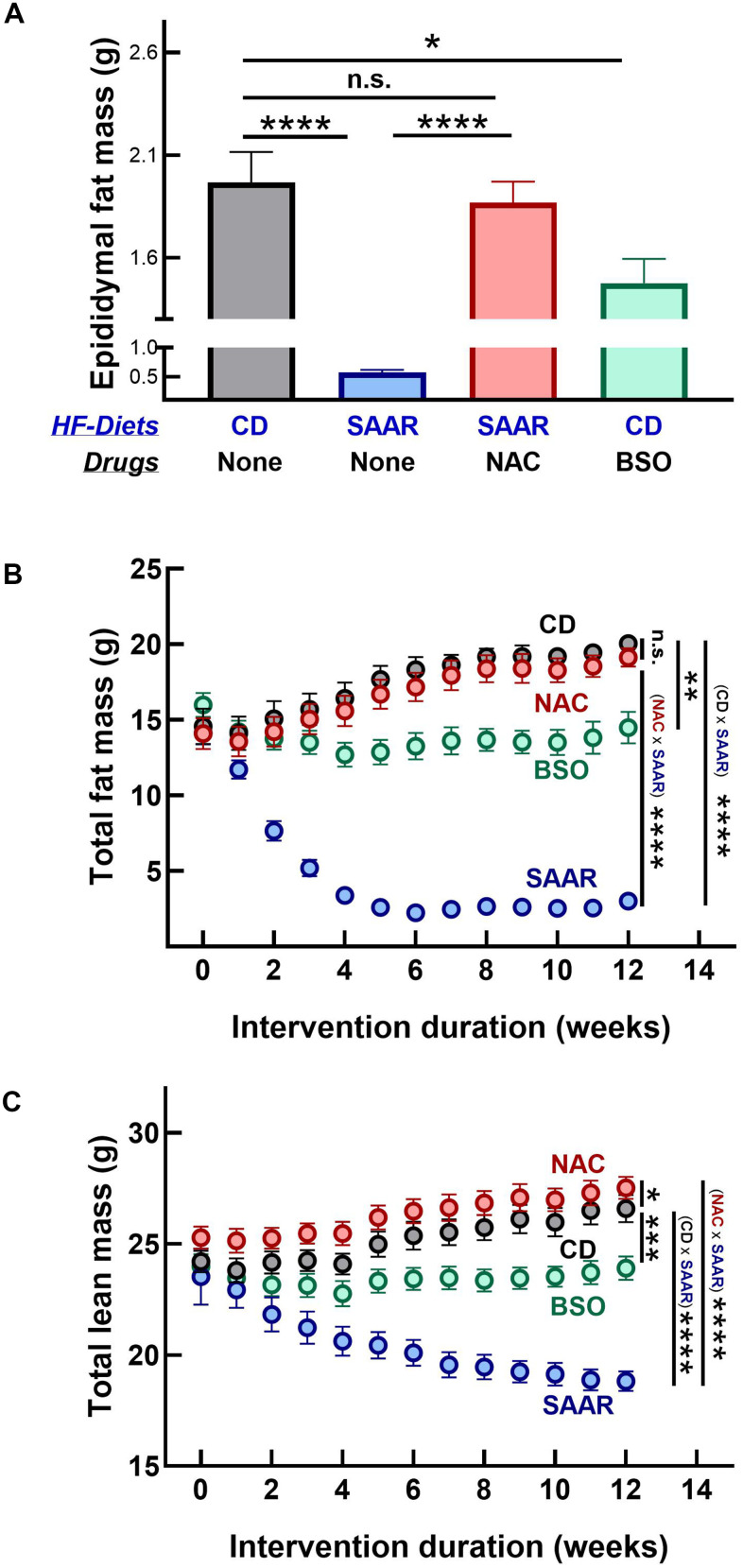
**BSO prevents fat mass gain without affecting lean mass.** The SAAR diet and BSO lowered epididymal fat mass (**A**). However, the SAAR diet exerted a strong effect on total body fat mass compared to the baseline values at week 0 (**B**); with a concomitant decrease in lean mass (**C**). Although it did not exert as strong an effect as the SAAR diet on total fat mass (**B**), BSO did not lower lean mass (**C**). On the other hand, NAC mice had values similar to those of CD mice (**A**–**C**). Note: Statistics and sample size are as mentioned in Figure 1.

Contrary to its similar effect on hepatic lipid droplet frequency, BSO did not decrease the adipocyte area in the epididymal fat depot, while the SAAR diet exerted a strong effect (SAAR – 37% of CD, Figure 4E, 4F). However, similar to the reversal of the SAAR-induced decrease in hepatic lipid droplet frequency, NAC reversed the decrease in adipocyte area in the epididymal fat depot (Figure 4E, 4F). While BSO did not affect the adipocyte area in the epididymal fat, it decreased the epididymal fat weight, although to a lesser extent than the SAAR diet (BSO-75% of CD, SAAR-29% of CD, Figure 5A). BSO decreased not just the epididymal fat depot weight, but also total body fat mass compared to CD mice (72% of CD, Figure 5B). The SAAR diet exerted a rapid and much stronger effect on the body fat mass than BSO (15% of CD). The time-related effects of the SAAR diet and BSO on body fat mass were similar to their effects on total body weight (Figures 1B, 5B), indicating that a significant portion of changes in body weight were due to changes in fat mass. The effect of BSO and the SAAR diet on body composition differed, at least in two aspects. Mice on the SAAR diet had much lower total fat mass after the intervention than at the baseline (21% of baseline, Figure 5B), while it remained similar in BSO mice. Notably, BSO did not decrease the total lean mass compared to the baseline, while the SAAR diet decreased the total lean mass by 80% of the lean mass at the baseline (Figure 5C). On the other hand, mice on NAC accrued fat mass and lean mass at the same rate as the CD mice (Figure 5B, 5C).

**Figure 6:**
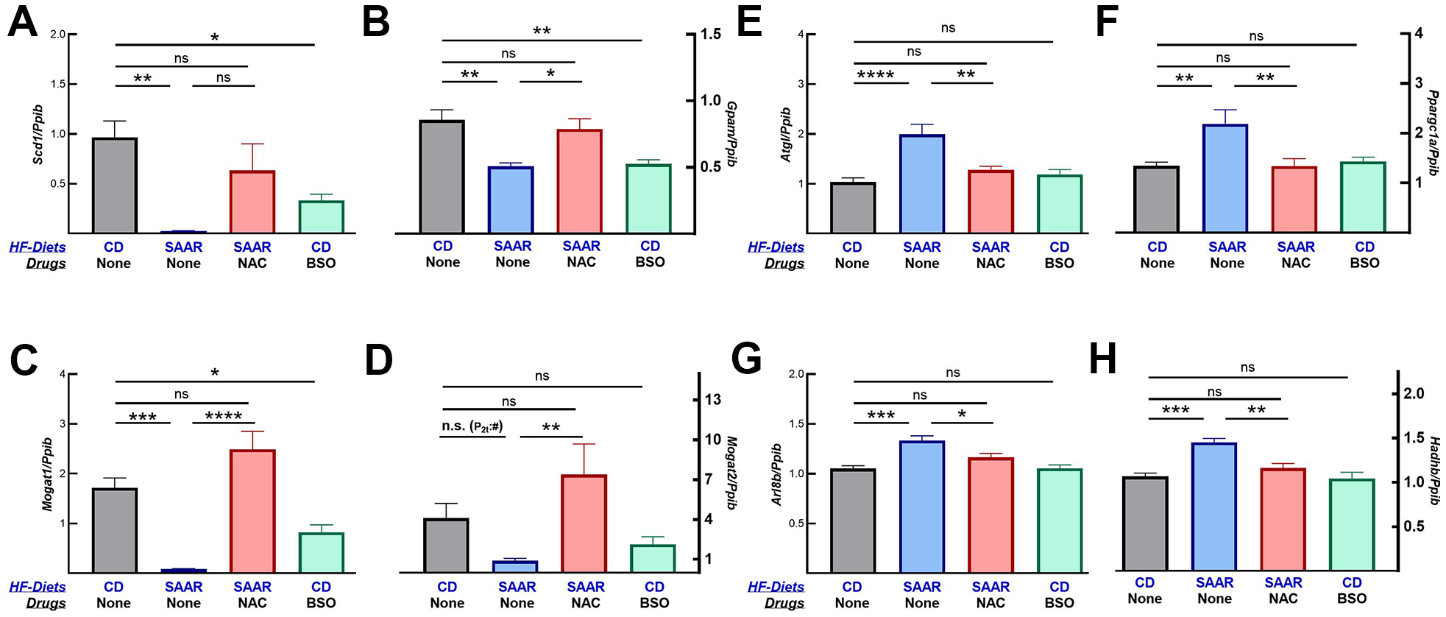
**BSO decreases the mRNA expression of lipogenic but not lipolytic genes.** Similar to the SAAR diet, BSO decreased the mRNA expression of lipogenic genes, including *Scd1* (**A**), *Gpam* (**B**), *Mogat1* (**C**), and *Mogat2* (**D**). The SAAR diet, but not BSO, increased the mRNA expression of lipolytic genes like *Atgl* (**E**), *Ppargc1a* (**F**), *Arl8b* (**G**), and *Hadhb* (**H**). NAC reversed the effects of the SAAR diet on all these genes. Note: Statistical methods are similar to those in Figure 2. Sample size is as mentioned in Figure 1.

Changes in body composition indicate that BSO might only prevent further weight gain while the SAAR diet induces weight loss. To investigate if our assumption was valid, we quantified the mRNA of proteins involved in lipogenesis and lipolysis. mRNA data show that both the SAAR diet and BSO decreased the expression of lipogenic genes (*Scd1*, *Gpam*, *Mogat1*, and *Mogat2*, Figure 6A–6D). In contrast, only the SAAR diet increased the expression of lipolytic genes (*Atgl*, *Ppargc1a*, *Arl8b*, and *Hadhb*, Figure 6E–6H). Like its effect on the rest of the markers, NAC reversed the SAAR-induced expression of all these genes (Figure 6).

### The SAAR diet and BSO induce similar metabolomic changes in the kidneys

**Figure 7:**
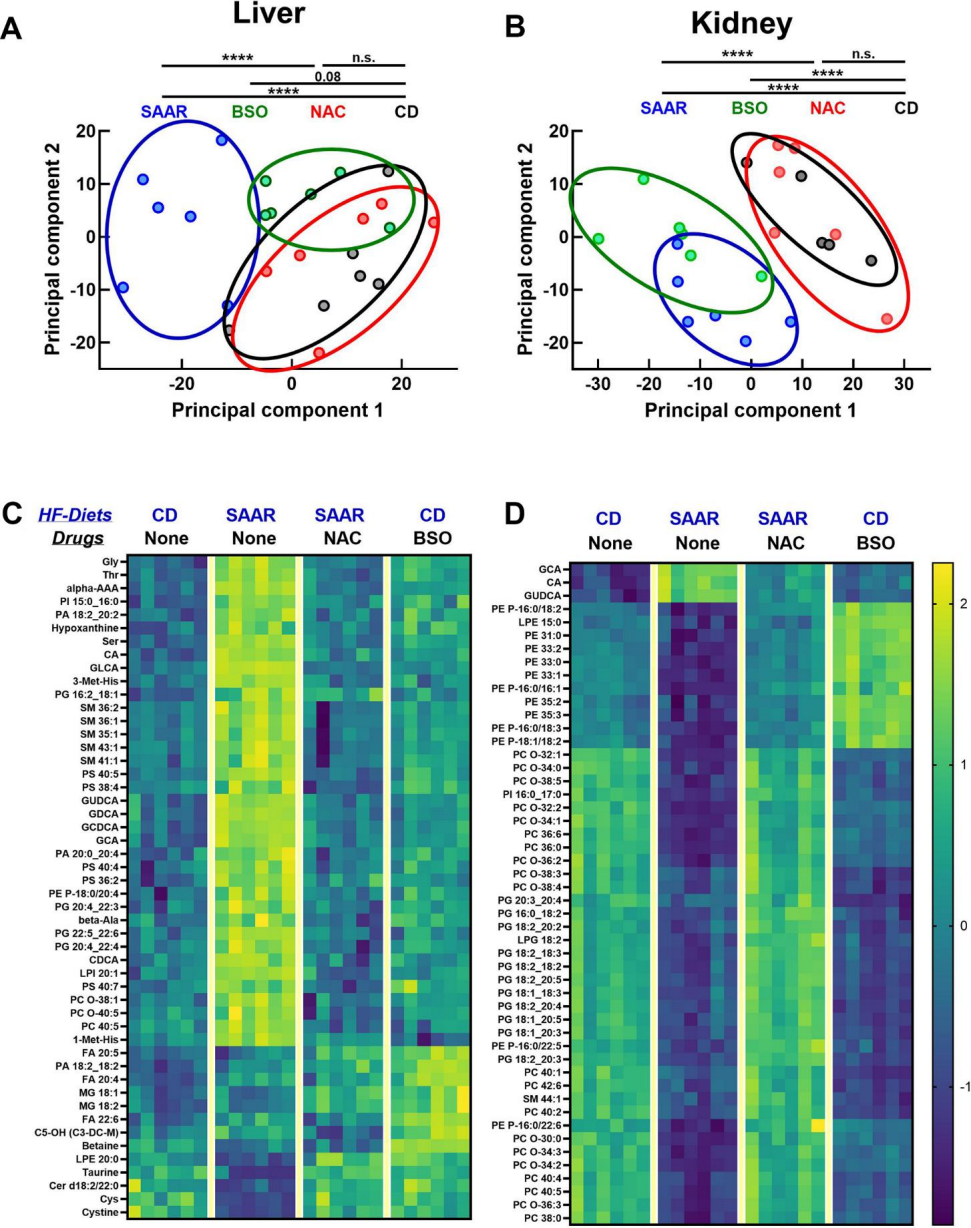
**BSO and the SAAR diet induce similar metabolomic profiles in the kidneys.** Metabolomics analyses revealed that the SAAR diet induces unique changes in the liver, which were reversed by NAC supplementation (overlap of NAC and CD clusters). (**A**) Although the BSO-induced changes appear as a cluster separated from CD, the statistical significance was on the borderline. However, BSO-induced changes in the kidney overlapped the SAAR-induced changes, while clusters from the NAC and CD groups overlap, indicating the reversal of SAAR diet-induced changes (**B**). Heat maps of the metabolites with the most significant changes in the liver and kidney are shown in (**C**, **D**).

We used targeted metabolomics to understand the broader metabolic impact of BSO and its similarities with the SAAR diet. Principal component analysis (PCA) revealed three clusters in the liver: the first belonged to the CD diet and NAC, the second to the SAAR diet, and the third to BSO (Figure 7A). PCA data from kidneys clustered into two distinct patterns, consisting of CD and NAC in the first one, and the SAAR diet and BSO in the second one (Figure 7B). The closer clustering of the BSO group with the SAAR diet in the kidneys than in the liver was concordant with the stronger effect on tGSH in kidneys than in the liver. Consistent with the trends in the PCA, the heatmaps of the top fifty metabolites with the most significant P-values show that changes in the SAAR diet and BSO are more similar in the kidneys than in the livers (Figure 7C, 7D). Many of these top fifty compounds were amino acids, bile acids, and different species of lipids. Overall, the data show that the BSO-induced metabolomic changes in the kidneys are similar to those in the SAAR diet.

## DISCUSSION

Failure to translate laboratory interventions is a significant hurdle to improving human healthspan. The strong anti-obesity effects induced by the SAAR diet in rodents were not replicable to the same extent in humans. This study demonstrates that the SAAR-induced lean phenotype can be recapitulated in mice fed a sulfur amino acid-replete diet by pharmacologically lowering GSH. Data also indicate the involvement of the Nrf2/Phgdh pathway, a putative mechanism of the SAAR-induced lean phenotype. Like the SAAR diet, BSO decreased the hepatic lipid droplet frequency, epididymal fat depot weights, and total fat mass. At the dose we used (30 mM), the effect of BSO on the decrease in fat mass (72% of CD) was lower than that of the SAAR diet (15% of CD). However, it is noteworthy that, unlike the SAAR diet, which decreased not just fat mass but also lean mass, BSO decreased only fat mass. Plasma markers of liver and kidney damage, AST, ALT, and cystatin-C show that the continuous administration of BSO was not toxic (Supplementary Figure 1). The concomitant reversal of the SAAR-induced lean phenotype and Nrf2 pathway by NAC indicates that low GSH and/or its precursor cysteine, rather than low methionine, are causally associated with the SAAR-induced lean phenotype. We propose BSO and other compounds that limit GSH biosynthesis as anti-obesity candidates in humans.

GSH, well-known for its antioxidant properties, is essential for many other functions, including cell metabolism [36]. GSH synthesis is regulated by glutamate-cysteine ligase, a heterodimer of the catalytic and modifier subunits, coded by *Gclc* and *Gclm*, respectively. Studies in genetic mouse models report that knocking out *Gclm* renders mice on a high-fat diet unable to utilize dietary fat for metabolic needs despite normal absorption from the gut. As a result, these mice had lower liver fat, total body fat, and body weight gain [37]. These reports align with BSO-induced changes in our study, including lower tissue GSH, higher Nrf2 expression, and downstream effects, including lower lipid droplet frequency, epididymal fat, and total body fat mass (Figure 5). In another study, liver-specific knockdown of *Gclc* in adult mice resulted in Nrf2 induction and anti-obesity phenotypes, including lower triglycerides and lipogenic gene expression [38]. Interestingly, the same study reported that Nrf2 cannot repress lipogenic genes unless its induction by *Gclc* knockdown is strong. This implies that only interventions that decrease GSH to concentrations below a certain threshold can repress liver lipogenesis. Such threshold-dependent effects of GSH were reported for various cellular processes, including gluconeogenesis, lipid peroxidation, and detoxification [39–41].

The SAAR diet and BSO exerted tissue-specific effects on GSH, Nrf2, and Phgdh. While both interventions lowered hepatic and renal GSH levels, their effects on Nrf2 and Phgdh were exclusive to one tissue. This tissue-specific effect could be due to the low threshold requirement. The magnitude of GSH decrease was higher in the livers than in the kidneys of the SAAR mice, while the vice versa was true for BSO mice (p < 0.0001). Consequently, the SAAR diet upregulated Nrf2 and Phgdh exclusively in the liver, whereas BSO triggered these responses only in the kidney. One possible reason for the high sensitivity of kidneys to BSO could be the method of administration. Prior research reported higher sensitivity of kidneys than the liver to BSO administered in water and that these two tissues respond differently even to intraperitoneal injection of BSO [42, 43]. Furthermore, it is well-established that hepatic GSH concentrations fluctuate significantly throughout the day, whereas renal GSH levels remain relatively stable [44]. It is also known that the Nrf2 expression itself fluctuates due to circadian rhythms [45–47]. A combination of these factors could contribute to the apparent lack of BSO effect on hepatic Nrf2 and Phgdh.

Regardless of the changes in Nrf2 and Phgdh, BSO increased hepatic serine concentrations. Higher hepatic serine could result from increased synthesis or decreased consumption during cysteine synthesis. During transsulfuration, serine conjugates with homocysteine to synthesize cystathionine, which is later converted to cysteine. Higher hepatic serine in BSO mice could be due to decreased transsulfuration, as indicated by lower cysteine concentrations (Figures 2A, 3C). From our study, we cannot conclude whether the higher hepatic serine in BSO mice resulted from the increased hepatic synthesis, decreased consumption, or increased uptake of the plasma serine sourced from the kidney. Regardless of the cause of higher hepatic serine, it was reported that an increase in renal serine synthesis and exogenous serine supplementation lowers hepatic lipogenesis and lipid droplet frequency [35]. Unlike the Nrf2 and Phgdh gene expression, which are subject to diurnal fluctuations and prandial state at the time of the sacrifice, hepatic lipid droplet frequency is relatively stable to diurnal fluctuations. It is a better marker to represent the anti-adiposity effect of BSO [48]. Since BSO decreased the hepatic lipid droplet frequency, we investigated its effect on mRNA expression of the genes involved in lipid droplet turnover (*Cidea*, *Cidec*, and *Arl8b*) and lipid metabolism (*Scd1*, *Fasn*, *Atgl*, and *Ppargc1a*). Data on mRNA expression are consistent with the morphological changes induced by BSO, i.e., its ability to prevent fat mass gain but not to induce fat mass loss. BSO decreased the expression of lipogenic genes, *Scd1* and *Fasn,* but did not affect lipolytic genes, *Atgl* and *Ppargc1a*. Similarly, it repressed the expression of proteins involved in forming lipid droplets (*Cidea* and *Cidec*) but did not affect the genes involved in their degradation (*Arl8b*). On the contrary, the SAAR diet had a broader effect on lipid metabolism. It favorably altered the expression of genes involved in lipogenesis (*Scd1*, *Gpam*, *Mogat1*, and *Mogat2*), lipolysis (*Atgl*, *Ppargc1a*, *Arl8*, and *Hadhb*), and lipid droplet formation (*Cidea* and *Cidec*). These widespread effects of the SAAR diet not only prevented the fat mass gain but also induced a robust loss of fat mass. Whether an increase in BSO dose would result in effects as strong and as broad as those induced by the SAAR diet and if a lower extent of SAAR would limit its effects to prevention of lipogenesis, remains to be explored.

Findings from the NAC group suggest that higher hepatic GSH abrogates the SAAR-induced lean phenotype. Since the NAC supplementation increased both hepatic GSH and cysteine, it is reasonable to speculate a role for cysteine in the NAC-induced reversal. While individual amino acids are reported to play signaling roles, much of the existing literature suggests that GSH, rather than cysteine, might be the cause [49, 50]. For instance, consistent with our hypothesis of the anti-adiposity effects of a low-GSH/Nrf2/Phgdh pathway, *Gclc* knockout mice with lower hepatic GSH had lower hepatic lipogenesis than the wild-type mice, despite both having similar cysteine concentrations [38]. Regardless of the hepatic cysteine concentration, multiple GSH-depleting agents induce Nrf2, which represses *Scd1*, an enzyme that regulates triglyceride synthesis [38, 51–53]. While these data strongly suggest that the anti-obesity effects of BSO were due to low tGSH, additional studies with simultaneous treatment of obese mice with cysteine and BSO might provide further evidence.

Besides upregulating serine biosynthesis, low-GSH might induce lean phenotype through other mechanisms. It was reported that a GSH decrement induces a thermogenic program in adipose tissue and improves insulin sensitivity [41–43]. Complementing these findings, other reports show that excess GSH leads to insulin resistance, induces adipocyte differentiation, and sustains the high NADPH required for fatty acid synthesis, implying that lower GSH might reverse all these processes [38, 54]. Other potential mechanisms include glutathionylation of proteins involved in intermediary metabolism, including glycolysis and the tricarboxylic acid cycle [55, 56]. The effect of low GSH on a wide range of mechanisms indicates that lowering GSH concentrations would be a good target for obesity.

It is commonly believed that lowering GSH levels, often achieved through BSO treatment, can have harmful effects. However, emerging evidence indicates that disproportionately high levels of cellular reducing capacity, driven by increased concentrations of GSH and other small molecules such as NADH and NADPH, leads to reductive stress, a condition associated with metabolic disorders such as obesity [57, 58]. Our current and previous studies suggest that BSO treatment might be beneficial in the context of diabetes and obesity [22]. Based on prior literature, one would expect BSO to induce hepatic damage and increase plasma ALT. However, in our study, BSO decreased the hepatic damage caused by the high-fat diet, indicating its therapeutic role (Supplementary Figure 1). Previous studies reported sexual dimorphism of GCL sensitivity to BSO; females were more sensitive than males [59]. Based on these reports, we speculate that females might be more sensitive to the BSO-induced lean phenotype than males; however, higher sensitivity also implies higher toxicity [59]. The beneficial and detrimental effects of BSO are highly context- and dose-dependent and should be evaluated on a case-by-case basis.

Since BSO was used in clinical trials against cancers, repurposing it for metabolic disease treatment in humans is relatively easy from a regulatory perspective [60, 61]. There is also considerable evidence from epidemiological studies indicating that pharmacological lowering of GSH might be beneficial. Total GSH concentrations and the ratio of reduced GSH to oxidized GSH were higher in overweight and obese adolescents compared to normal-weight individuals [62]. Some population studies report a positive correlation of metabolic disease markers with plasma cysteine but not with plasma GSH [63, 64]. Findings from such studies should be interpreted by considering the differential distribution of the two thiol compounds. Most of the GSH in blood occurs in erythrocytes. In contrast, most of the cysteine occurs in plasma, resulting in approximately a twenty-fold higher concentration of cysteine than GSH in plasma [65, 66]. Since plasma GSH is a poor indicator of tissue GSH concentrations, findings from epidemiological studies should be cautiously interpreted. Preclinical and clinical studies report that most anti-obesity interventions are associated with decreased food intake and muscle mass [67, 68]. In our study, BSO prevented fat mass gain without affecting lean mass or decreasing food intake. In addition, we have recently demonstrated that BSO also improves glucose tolerance in mice affected with proinsulin misfolding, indicating that BSO might improve other aspects of metabolic diseases [22]. Overall, additional studies in rodents and pilot clinical trials to investigate the therapeutic effects of BSO against metabolic diseases are highly warranted.

Since GSH plays multifaceted roles in cellular physiology, it will likely affect overall healthspan and lifespan. By attenuating GSH synthesis, BSO can trigger the expression of Nrf2, which is associated with multiple aging hallmarks, including cellular senescence, proteostasis, genomic stability, and telomere length [69–71]. Studies in *C. elegans* support this hypothesis. Inhibition of GSH synthesis in *C. elegans* using RNAi against *gcs1* (an ortholog of the mammalian *Gclc*) increased lifespan [72]. This same study also found that *skn1*, the worm ortholog of Nrf2 in mammals, was crucial for the *gcs-1*/RNAi-induced lifespan extension and that multiple genes under the transcriptional control of *skn1* were induced. Another study shows that increasing GSH concentration can have detrimental effects on healthspan and lifespan. Feeding *C. elegans* either GSH or NAC accelerated aging in a dose-dependent manner [73]. This study also reported that both compounds inhibited *skn1* expression, suggesting its role in modulating *C. elegans* lifespan. We quantified a few plasma markers from our study to investigate if BSO exerts any effects on the healthspan, in mammals. Data show that just like the SAAR diet, BSO decreased plasma insulin and IGF-1 (Supplementary Figure 2). Unlike the SAAR diet, BSO did not increase plasma adiponectin. Overall, data from our study lay the foundation for lifespan extension studies in *C. elegans* and mammalian models using BSO.

## Supplementary Material

Supplementary Figures

Supplementary Tables
