# Supplementary material for: Pharmacological recapitulation of the lean phenotype induced by the lifespan-extending sulfur amino acid-restricted diet
Source: Aging (Albany NY). 2025 Apr 7;17(4):960–81. doi: 10.18632/aging.206237 (PMC12074818; doi:10.18632/aging.206237)
Supplement: Supplementary Figures [file aging-17-206237-s001.pdf]

SUPPLEMENTARY FIGURES

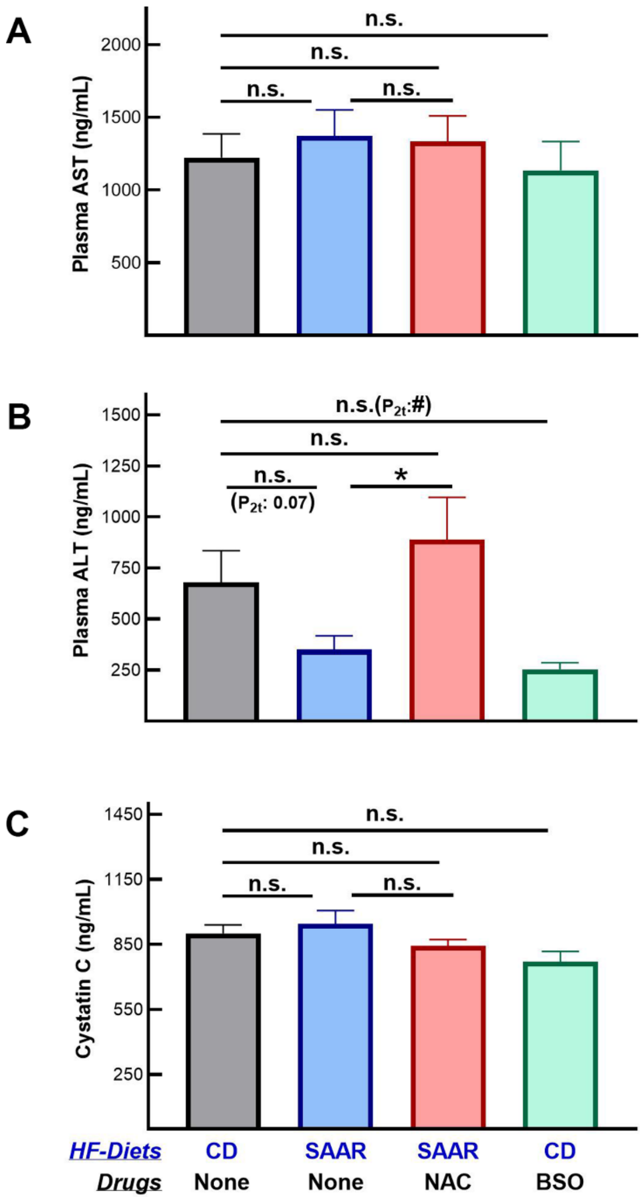

Supplementary Figure 1. Long-term administration of BSO does not induce adverse effects. Thirteen weeks of continuous BSO administration (30 mM in water) did not exert adverse effects, as indicated by no increase in plasma markers of liver and kidney damage, aspartate transaminase (**A**, AST), alanine transaminase (**B**, ALT), and cystatin C (**C**). Of note, the trend of lower levels of ALT in the SAAR and BSO groups indicates that both interventions might reduce the hepatic damage induced by the high-fat diet. Statistics and sample size are similar to those in Figure 2.

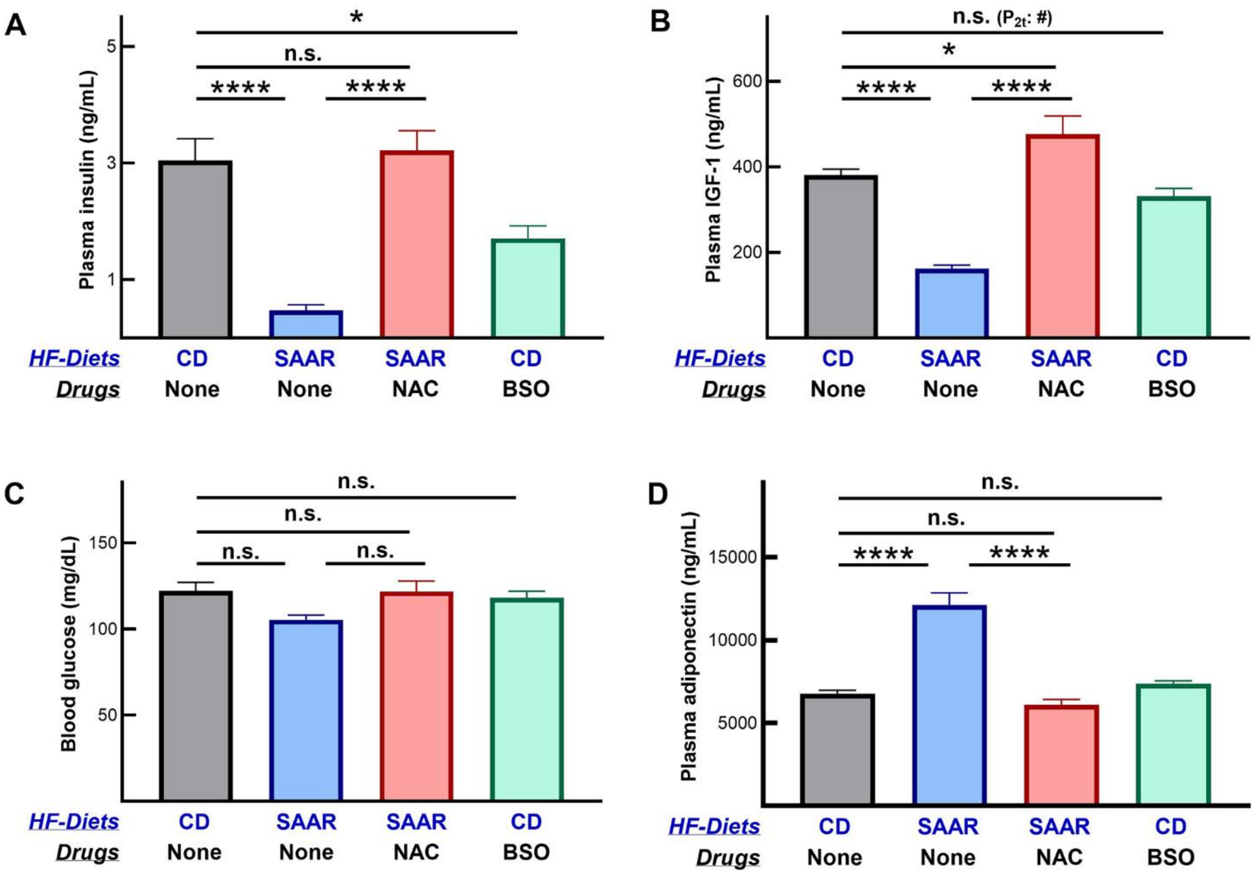

Supplementary Figure 2. BSO decreases plasma insulin and IGF-1. Like the SAAR diet, BSO decreased plasma insulin (**A**) and IGF-1 (**B**); neither interventions decreased random blood glucose (**C**). The SAAR diet, but not BSO increased adiponectin (**D**). NAC reversed all SAAR-induced changes in plasma hormones.
