# Supplementary material for: Pharmacological recapitulation of the lean phenotype induced by the lifespan-extending sulfur amino acid-restricted diet
Source: Aging (Albany NY). 2025 Apr 7;17(4):960–81. doi: 10.18632/aging.206237 (PMC12074818; doi:10.18632/aging.206237)
Supplement: Supplementary Tables [file aging-17-206237-s002.pdf]

SUPPLEMENTARY TABLES

Supplementary Table 1. Dietary composition.

Ingredient	CD (A14032002)[a]	SAAR (A14032001)[a]
	g % (kcal%)	g % (kcal%)
Protein	17 (13)	17 (13)
Carbohydrate	36 (28)	36 (28)
Fat	35 (60)	35 (60)
Kcal/g[b]	5.3	5.3
L-Arginine	1.48 (1.13)	1.48 (1.13)
L-Histidine-HCl-H_2O	0.44 (0.33)	0.44 (0.33)
L-Isoleucine	1.09 (0.83)	1.09 (0.83)
L-Leucine	1.47 (1.1)	1.47 (1.1)
L-Lysine	1.91 (1.45)	1.91 (1.45)
**DL-Methionine**	**0.86 (0.65)**	**0.12 (0.1)**
**L-Cysteine**	**0 (0)**	**0 (0)**
L-Phenylalanine	1.53 (1.15)	1.53 (1.15)
L-Threonine	1.09 (0.83)	1.09 (0.83)
L-Tryptophan	0.24 (0.18)	0.24 (0.18)
L-Valine	1.09 (0.83)	1.09 (0.83)
**L-Glutamic Acid**	**2.7 (2.05)**	**3.43 (2.6)**
Glycine	3.08 (2.33)	3.08 (2.33)
Maltodextrin	8.68 (6.55)	8.68 (6.55)
Dextrose	6.62 (5)	6.62 (5)
Sucrose	19.85 (15)	19.85 (15)
Cellulose	6.62 (0)	6.62 (0)
**Lard**	**28.98 (49.28)**	**28.98 (49.28)**
**Corn Oil**	**6.09 (10.35)**	**6.09 (10.35)**
Mineral Mix S10001	4.63 (0)	4.63 (0)
Vitamin Mix V10001	1.32 (1)	1.32 (1)
Choline Bitartrate	0.26 (0)	0.26 (0)

[a]Research Diets catalog number.
[b]Energy density was determined using Atwater factor system.

Supplementary Table 2. Technical details of the Western blot procedure.

Antibody	Vendor	Catalog No.	Host species	Antibody dilutions
**Primary Antibodies**				
Nrf2	Cell Signaling Technology	20733	Rabbit	1:1000 in 5% BSA[a]
Phgdh	Cell Signaling Technology	13428	Rabbit	1:1000 in 5% milk[a]
β-Actin	Sigma	A5441	Mouse	1:15000 in 5% milk[a]
Vinculin	Proteintech	66305-1-Ig	Mouse	1:10000 in 5% milk[a]
**Secondary Antibodies**				
Anti-Rabbit-HRP	Cell Signaling Technology	7074	Goat	1:4000 in 5% milk, 1h[b]
Anti-Mouse-HRP	Bio-Rad	170-6516	Goat	1:20000 in 5% milk, 30 min[b]

[a]All incubations were performed overnight at 4° C.
[b]All incubations were performed at room temperature.

Supplementary Table 3. TaqMan assays used in real-time PCR.

Gene name	Assay ID
Scd1	Mm00772290_m1
Gpam	Mm00833328_m1
Mogat1	Mm00503357_m1
Mogat2	Mm00624192_m1
Atgl	Mm00503040_m1
Ppargc1a	Mm01208835_m1
Arl8b	Mm00482600_m1
Hadhb	Mm00695255_g1
Ppib	Mm00478295_m1

Supplementary Table 4. Details of ELISA kits used in the study.

Enzyme name	ELISA kit Cat. No.	Vendor
Aspartate aminotransferase	Mouse AST ELISA Kit (Aspartate Aminotransferase, ab263882)	Abcam
Alanine transaminase	Mouse ALT ELISA Kit (ab282882)	Abcam
Cystatin C	Mouse Cystatin C (ab201280)	Abcam
Insulin	Mouse Ultrasensitive Insulin ELISA (80-INSMSU-E01)	ALPCO
Insulin-like Growth Factor 1	Mouse/Rat IGF-1 Quantitative ELISA Kit (MG100)	R&D Systems
Adiponectin	Mouse Adiponectin/Acrp30 Quantitative ELISA Kit (MRP300)	R&D Systems
